# *Leisingera* sp. JC1, a Bacterial Isolate from Hawaiian Bobtail Squid Eggs, Produces Indigoidine and Differentially Inhibits Vibrios

**DOI:** 10.3389/fmicb.2016.01342

**Published:** 2016-09-08

**Authors:** Samantha M. Gromek, Andrea M. Suria, Matthew S. Fullmer, Jillian L. Garcia, Johann Peter Gogarten, Spencer V. Nyholm, Marcy J. Balunas

**Affiliations:** ^1^Division of Medicinal Chemistry, Department of Pharmaceutical Sciences, University of ConnecticutStorrs, CT, USA; ^2^Department of Molecular and Cell Biology, University of ConnecticutStorrs, CT, USA; ^3^Institute for Systems Genomics, University of ConnecticutStorrs, CT, USA

**Keywords:** symbiosis, *Euprymna*, roseobacter, *Rhodobacteraceae*, indigoidine, *Leisingera*, DART-MS, secondary metabolite regulation

## Abstract

Female members of many cephalopod species house a bacterial consortium in the accessory nidamental gland (ANG), part of the reproductive system. These bacteria are deposited into eggs that are then laid in the environment where they must develop unprotected from predation, pathogens, and fouling. In this study, we characterized the genome and secondary metabolite production of *Leisingera* sp. JC1, a member of the roseobacter clade (*Rhodobacteraceae*) of *Alphaproteobacteria* isolated from the jelly coat of eggs from the Hawaiian bobtail squid, *Euprymna scolopes*. Whole genome sequencing and MLSA analysis revealed that *Leisingera* sp. JC1 falls within a group of roseobacters associated with squid ANGs. Genome and biochemical analyses revealed the potential for and production of a number of secondary metabolites, including siderophores and acyl-homoserine lactones involved with quorum sensing. The complete biosynthetic gene cluster for the pigment indigoidine was detected in the genome and mass spectrometry confirmed the production of this compound. Furthermore, we investigated the production of indigoidine under co-culture conditions with *Vibrio fischeri*, the light organ symbiont of *E. scolopes*, and with other vibrios. Finally, both *Leisingera* sp. JC1 and secondary metabolite extracts of this strain had differential antimicrobial activity against a number of marine vibrios, suggesting that *Leisingera* sp. JC1 may play a role in host defense against other marine bacteria either in the eggs and/or ANG. These data also suggest that indigoidine may be partially, but not wholly, responsible for the antimicrobial activity of this squid-associated bacterium.

## Introduction

It is becoming increasingly evident that many animals and plants use compounds produced by symbiotic bacteria for protection against pathogens and other fouling organisms (reviewed in Flórez et al., 2015). In marine and aquatic environments a number of invertebrates (including sponges, tunicates, bryozoans, and molluscs) host microorganisms that produce compounds used for such protection. These groups have served as an important source for studying defensive symbioses and for the discovery of novel bioactive natural products (see example in Schmidt and Donia, 2010).

Among molluscs, one common yet poorly understood animal–bacterial association occurs between members of squid and cuttlefish species and bacterial consortia that reside within a reproductive gland of female hosts called the accessory nidamental gland (ANG; Kaufman et al., 1998; Grigioni et al., 2000; Barbieri et al., 2001; Pichon et al., 2005; Collins et al., 2012). This organ harbors a dense consortium of bacteria housed in epithelium-lined tubules that are attached to the nidamental gland, the organ that secretes the jelly coat (JC) surrounding fertilized eggs. Bacteria from the ANG are deposited into the JC where they have been hypothesized to help protect developing eggs from fouling microorganisms, pathogens, and/or predation (Barbieri et al., 1997, 2001; Collins et al., 2012, 2015).

The Hawaiian bobtail squid, *Euprymna scolopes*, has been used as a model organism to study bacteria–host interactions, mainly due to the host’s relationship with the bioluminescent bacterium *Vibrio fischeri* (McFall-Ngai, 2014). Recent studies have also focused on a second association found within the ANG of this species (Collins and Nyholm, 2011; Collins et al., 2012, 2015). These studies demonstrated that the ANG consortium in *E. scolopes* is dominated by members of the *Rhodobacteraceae* (roseobacters) within the *Alphaproteobacteria*, a common group of marine bacteria. A number of roseobacter-clade organisms are known to produce unique antimicrobial molecules and other secondary metabolites. For example, the antibiotic tropodithietic acid (TDA) and the algicidal roseobacticides are produced by *Phaeobacter* species and the antibacterial compound indigoidine is produced by *Leisingera* (formerly *Phaeobacter*) sp. Y4I (Geng et al., 2008; Seyedsayamdost et al., 2011; Cude et al., 2012). Most of these studies have focused on either free-living or plankton-associated roseobacters and the potential antimicrobial activity of the ANG strains has not been explored. A study that analyzed the genomes of 13 ANG roseobacter strains from *E. scolopes* did reveal the potential for secondary metabolite production (Collins et al., 2015) and *Gammaproteobacteria* from the ANG of another squid species have been shown to inhibit other bacteria (Barbieri et al., 1997).

In this study, we characterized the genome and secondary metabolite production of a new bacterial strain, *Leisingera* sp. JC1, isolated from the JC of *E. scolopes* squid eggs. Whole genome sequencing and biochemical analyses revealed the potential for and production of a number of secondary metabolites, including siderophores and acyl-homoserine lactones involved with quorum sensing. The complete indigoidine biosynthetic gene cluster was detected in the genome and mass spectrometry confirmed the production of this compound. Furthermore, we investigated the regulation of indigoidine under co-culture conditions with *V. fischeri*, the light organ symbiont. Finally, both *Leisingera* sp. JC1 and extracts from this strain exhibited differential antimicrobial activity against a number of marine vibrios, suggesting that indigoidine may be partially, but not wholly, responsible for the antimicrobial activity of this squid-associated bacterium.

## Materials and Methods

### Bacterial Isolation

Hawaiian bobtail squid, *E. scolopes*, were obtained from sand flats in Oahu (Maunalua Bay, 21°16′51.42″ N, 157°43′33.07″ W), Hawaii and maintained in aquaria as previously described (Schleicher and Nyholm, 2011). Eggs laid in captivity from one adult female were collected, flash frozen on the 11th day of development, and stored at -80°C. Ten eggs were thawed for bacterial isolation and their outer capsules and embryos were removed and discarded with sterile forceps. The JCs were isolated, surface sterilized with 70% ethanol, and rinsed with filter-sterilized squid Ringers (FSSR, 530 mM NaCl, 25 mM MgCl_2_, 10 mM CaCl_2_, 20 mM HEPES, pH = 7.5). The 10 JCs were pooled and homogenized in FSSR, then serially diluted and plated on seawater tryptone (SWT) medium (5 g/L tryptone, 3 g/L yeast extract, 3 mL/L glycerol, 700 mL/L Instant Ocean sea salts, 15 g/L agar, 300 mL/L DI water). *Leisingera* sp. JC1 colonies appeared dark blue on this medium and were streaked to isolation.

### Genomic Sequencing and Analysis

Genomic DNA was extracted using the MasterPure DNA Purification kit (Epicentre, Madison, WI, USA) from an overnight liquid culture of *Leisingera* sp. JC1 grown shaking at 30°C in SWT. DNA was quantified using a Qubit 2.0 fluorometer (Life Technologies, Agawam, MA, USA) and checked for quality on a 1% agarose gel and using a NanoDrop 1000 spectrophotometer (Thermo Scientific, Agawam, MA, USA). A paired end library was prepared from 1 ng of genomic DNA using the Nextera XT DNA library kit (Illumina, Inc., San Diego, CA, USA) and quantified using the Qubit fluorometer and bioanalyzer (Agilent Technologies, Santa Clara, CA, USA). The library was sequenced on an Illumina MiSeq sequencer using 2 bp × 250 bp reads at the Microbial Analysis Resources and Services (MARS) facility at the University of Connecticut (Storrs, CT, USA).

Reads were trimmed using the CLC Genomic Workbench (Qiagen, Hilden, Germany) and a draft genome was assembled using the A5 assembler (Tritt et al., 2012). Coverage was determined by mapping trimmed reads to the draft genome assembly using CLC Genomic Workbench. The genome was annotated using the Rapid Annotation using Subsystem Technology (RAST, Aziz et al., 2008)^1^ server and analyzed with the Antibiotic and Secondary Metabolite Analysis Shell 3.0 (antiSMASH, Weber et al., 2015)^2^ for potential secondary metabolite biosynthesis gene clusters. The draft genome assembly has been deposited in DDBJ/EMBL/GenBank under accession LYUZ00000000. The version described in this paper is version LYUZ01000000.

### Taxonomic Analysis and Whole Genome Comparison

Initial 16S identity suggested JC1 belonged to the genus *Leisingera* (data not shown). To validate this conclusion and to evaluate its relationship to the previously sequenced ANG isolates, a further taxonomic analysis was undertaken that used 17 previously described *Leisingera* genomes (Collins et al., 2015). A 33 gene multilocus sequence analysis was carried out following the methodology described in Collins et al. (2015). After generating alignments for each of the 33 genes using MUSCLE (Edgar, 2004), a concatenated alignment was generated using in-house python scripts. An optimal model of evolution was determined using the Akaike information criterion with correction for small sample size as implemented in jModelTest v2.1.4 (Darriba et al., 2012). The best-fitting model reported was GTR + Gamma estimation + Invariable site estimation. A maximum-likelihood (ML) phylogeny was generated from the concatenated multi-sequence alignment using PhyML v3.0_360-500M (Guindon et al., 2010). PhyML parameters consisted of GTR model, estimated p-invar, four substitution rate categories, estimated gamma distribution, sub-tree pruning and regrafting enabled with 100 bootstrap replicates. In addition to the maximum-likelihood analysis, a Bayesian inference analysis was also conducted using MrBayes v3.2.4 x64 (Ronquist et al., 2012). A mixed model with gamma estimation and invariable sites was used. The mixed model settled on a GTR submodel with only one parameter difference from the default GTR model with a posterior probability > 0.8. The standard GTR model accounted for the remainder of the model probability. The analysis used two cold chains with three heated chains each and ran for one million generations. After the run finished, convergence was assessed using average standard deviation of split frequencies of the cold chains, potential scale reduction factors of parameters, and minimum effective sample sizes of parameters. All criteria indicated the runs had converged.

Average nucleotide identity (ANI) was calculated using JSpecies 1.2.1 (Richter and Rosselló-Móra, 2009). The calculations were made using the MUMmer aligner with its default options. Contig files were generated for this analysis using the seqret function of the EMBOSS package (Rice et al., 2000). The reciprocal comparisons were averaged for reporting. Estimates of *in silico* DDH were made using the Genome-to-genome distance calculator 2.1 (Meier-Kolthoff et al., 2013) using the BLAST+ alignment method and the formula 2 algorithm outputs.

Select genomes were compared using the BLAST Ring Generator (BRIG) v1.0 (Alikhan et al., 2011). Default BLAST options were used. A whole genome alignment was generated using the Mauve program v2.3.1 (Darling et al., 2010). The progressiveMauve algorithm was used with default options.

### Homoserine Lactone Detection

Homoserine lactone (HSL) production was detected using a well-diffusion assay with the HSL-sensing bacterium *Agrobacterium tumefaciens* NTL4 pZLR4 (Cha et al., 1998) as previously described (Ravn et al., 2001; Collins et al., 2015). In brief, *A. tumefaciens* NTL4 was grown in 3 mL of LB with 30 μg/mL gentamicin for 24 h at 30°C. This culture was used to inoculate 50 mL of AB minimal media with 0.5% casamino acids and 0.5% glucose (Chilton et al., 1974), and allowed to grow for another 24 h at 30°C. This culture was used to inoculate 100 mL of AB minimal media to which 1.2% agar had been added and a final concentration of 0.5% casamino acids, 0.5% glucose, and 75 μg/mL 5-bromo-4-chloro-3-indolyl-β-D-galactopyranoside (X-gal) was added after autoclaving. The inoculated molten agar was allowed to solidify in Petri dishes and wells were cut into the media using a sterile borer.

*Leisingera* sp. JC1 and *Leisingera* sp. ANG1 were grown overnight at 30°C in 3 mL of SWT broth with 30 μM FeCl_3_, 0.5% glucose, and 0.5% casamino acids to induce HSL production. Cells were pelleted and the supernatant was collected and filtered through a 0.22 μm filter (Thermo Scientific, Agawam, MA, USA). Cell-free supernatant (60 μl) was added to the wells in the *A. tumefaciens* plates. The *N*-3-oxohexanoyl homoserine lactone standard was serially diluted and added to wells of an *A. tumefaciens* plate as a control and for semi-quantitative comparison. All plates were incubated at 28°C for 24 h before imaging.

### Siderophore Detection

To qualitatively detect siderophore production, *Leisingera* sp. JC1 was plated in triplicate on chrome azurol S (CAS) indicator agar, modified for marine bacteria as previously described (Whistler and Ruby, 2003), and incubated at 28°C for 24 h before imaging. Sequestration of iron from CAS causes a color change from blue to orange, indicating siderophore production.

### Detection of Indigoidine Biosynthesis Genes in JC1 Genomic DNA

To confirm the presence of indigoidine biosynthesis genes in JC1, genomic DNA was extracted and quantified as described for genomic sequencing above. Primers were designed (Supplemental Table S1) to amplify the *igiCDR* genes based on the draft genome assembly and using Primer3 software (Untergasser et al., 2012). PCR amplification was performed using the standard GoTaq Green Master Mix (Promega, Madison, WI, USA) protocol with 30 cycles and 55°C annealing temperature.

### *Leisingera* sp. JC1 Large Scale Culture

*Leisingera* sp. JC1 was cultured for extraction using SWT media (as described above except without addition of glycerol, delineated hereafter as SWT_ng_). A three step culturing process was employed to produce sufficient scale for secondary metabolite extraction, while ensuring that the bacterium was in late stationary phase for optimal production of secondary metabolites (Ruiz et al., 2010). First, small scale cultures were prepared by inoculating a JC1 colony into 5 mL of media in a 24 deep well plate, which was incubated for 3 days at room temperature while shaking at 200 rpm. Then, medium scale cultures were prepared by transferring 1.5 mL of the small scale cultures into 125 mL baffled flasks with 50 mL media, which were incubated for 3 days at room temperature while shaking at 125 rpm. Lastly, large scale cultures were prepared by transferring 15 mL of medium scale cultures into 1 L baffled flasks with 500 mL of media, which were incubated for 3 days at room temperature while shaking at 125 rpm.

### Extraction of *Leisingera* sp. JC1

All extraction solvents were ACS grade and purchased from Sigma Aldrich (St. Louis, MO, USA).

#### Normal Extraction

Diaion HP20 resin (Supelco, Bellefonte, PA, USA) was pre-washed by sequentially rinsing resin with methanol and Millipore water (EMD Millipore, Billerica, MA, USA). Large scale JC1 cultures were sonicated to lyse cells prior to addition of pre-washed Diaion HP20 resin (50 g, 10% w/v), followed by incubation for 24 h at room temperature while shaking at 125 rpm. Bacterial culture and resin were then filtered using a coarse glass frit filter and washed with Millipore water to remove aqueous media components. The resin and bacterial culture were then sequentially extracted with methanol, dichloromethane, and acetone (2 × 150 mL). Organic portions were combined, extracted with ethyl acetate to remove residual aqueous material, and concentrated.

#### Indigoidine Enriched Extraction

Because indigoidine is poorly soluble in water and most organic solvents, a second extraction protocol was utilized to prepare an indigoidine enriched extract following modified literature procedures (Yu et al., 2013). Briefly, large scale cultures were sonicated to lyse cells and transferred to centrifuge tubes. Cells were then separated from supernatant by low-speed centrifugation (850 *g* × 5 min; Beckman Coulter Avanti J-E Centrifuge, Brea, CA, USA). Supernatant was transferred to new tubes and subjected to high-speed centrifugation (21,000 *g* × 10 min) to obtain an indigoidine enriched pellet. The pellet was washed with methanol, transferred to a microcentrifuge tube, dried under N_2_ gas, and dissolved in dimethyl sulfoxide (DMSO).

### Detection of Indigoidine Production by *Leisingera* sp. JC1 via LC–MS

All HPLC grade solvents and reagents were purchased from Sigma-Aldrich. LC–MS data were collected on an Agilent ESI single quadrupole mass spectrometer coupled to an Agilent 1260 HPLC system with a G1311 quaternary pump, G1322 degasser, and a G1315 diode array detector (Agilent Technologies, Santa Clara, CA). A gradient elution was used from 10% methanol in H_2_O to 90% methanol in H_2_O over 25 min using an Agilent Eclipse XDB-C_18_ RP-HPLC column (4.6 mm × 150 mm, 5 μm) and a flow rate of 1 mL/min. Indigoidine enriched extracts were prepared at 5 mg/mL in DMSO. Indigoidine eluted at retention time (t_R_) 10.7 min in agreement with literature (Yu et al., 2013).

### Zone of Inhibition Assays

To observe inhibition of vibrio strains and ANG isolate strains by *Leisingera* sp. JC1 (Supplementary Table S3), a zone of inhibition (ZOI) assay was used. The vibrio strains *V. anguillarum* 775*, V. parahaemolyticus* KNH1*, V. fischeri* ES114, *V. harveyi* B392, and *Photobacterium leiognathi* KNH6 were grown for 2.5 h (to stationary phase) at 30°C in YTSS (4 g/L tryptone, 2.5 g/L yeast extract, 15 g/L Instant Ocean sea salts) broth and then serially diluted from 10^7^ to 10^4^ CFU/mL in YTSS broth to observe density dependent inhibition. Each dilution was plated in triplicate on YTSS agar using a sterile swab to form a lawn. All ANG isolates tested were grown overnight (~4 × 10^8^ CFU/mL) in SWT broth at 30°C and plated on SWT agar using a sterile swab to form a lawn. *Leisingera* sp. JC1 was grown overnight to a density of ~1 × 10^8^ CFU/mL in SWT when testing with ANG isolates and in YTSS when testing with vibrio strains. This overnight broth of *Leisingera* sp. JC1 was spotted (10 μL) on the surface of each lawn in quadruplicate. All plates were incubated at 28°C for 24 h before imaging and ZOI measurements around the *Leisingera* sp. JC1 colonies. SWT or YTSS broth (10 μL) were spotted on each lawn as media controls, and 10 μL of the overnight culture of *Leisingera* sp. JC1 was spotted in quadruplicate on SWT or YTSS agar without any bacterial lawns as a growth control.

To quantify inhibition, an average of three ZOI diameters were measured and an average of three diameters of the JC1 colonies were measured using ImageJ (Schneider et al., 2012). Due to slight variations in JC1 colony size across trials, the measurements were normalized by subtracting the average JC1 colony diameter from the average ZOI diameter. To determine if differences in ZOIs across lawn densities per organism were statistically significant, one-way ANOVAs were performed. If the results of the one-way ANOVA indicated statistically significant differences, multiple comparisons *post hoc* Tukey tests were performed to determine which lawn densities were significantly different.

### 96-Well Liquid Assays

*Leisingera* sp. JC1 extracts were tested for antibacterial activity against *V. fischeri* ES114*, V. anguillarum* 775, and *V. parahaemolyticus* KNH1. High throughput assays with these bacterial strains were developed based on similar assays with natural product extracts and human pathogens (Zgoda and Porter, 2001), including obtaining CFU counts and growth curves for each of the vibrio strains as well as determining proper incubation times and temperatures and finding appropriate controls. These assays were performed in 96-well plates (Corning Costar, Corning, NY, USA) with SWT media and incubated at 28°C while shaking at 200 rpm. The bacterial inocula were prepared by adding select colonies into 5 mL of media and adjusted to OD_600_ 0.1 (approximately 1–2 × 10^8^ CFU/mL as per Clinical and Laboratory Standards Institute, 2012). Colony forming unit (CFU) counts were manually confirmed to ensure accurate approximation for each vibrio strain.

Extracts were screened as previously described (Zgoda and Porter, 2001) with the following modifications. Briefly, master mix was prepared by addition of 1.6 mL adjusted vibrio inoculum, 7.84 mL sterile water, and 6.4 mL of SWT media. To each well, 198 μL of master mix was added with 2 μL of either positive control (chloramphenicol, final testing concentration 2.5 μg/mL), negative control (DMSO), or extract prepared in DMSO (screened at final concentration of 500 μg/mL; MIC performed using serial dilutions). Sterility control wells consisted of 98 μL sterile water, 100 μL of SWT media, and 2 μL of DMSO. All controls and samples were tested in technical triplicates with experiments repeated a minimum of three times to confirm results. Plates were read at 600 nm every 2 h from 0 to 10 h with a final reading at 24 h using a Synergy H1 Hybrid Reader (Biotek, Winooski, VT, USA). Results are given as percent control activity (PCA) calculated in comparison with DMSO, the negative control.

### Localization of Indigoidine Production by *Leisingera* sp. JC1 Using DART-MS

Direct analysis in real time-mass spectrometry (DART-MS) analysis was performed using a JEOL AccuTOF with DART ion source (IonSense, Inc., Saugus, MA, USA). High purity helium 5.0–6.0 grade (greater than 99.999% purity) was heated to 300°C and used for ionization. Five locations were selected on JC1 colonies in the presence or absence of *V. fischeri*, including (A) center of colony, (B) midpoint between center and edge of colony, (C) edge of colony, (D) ZOI (in the absence of *V. fischeri* sample was obtained from a point equidistant from colony edge), and (E) outside ZOI. At each location a sterile single use syringe needle (BD Medical, Franklin Lakes, NJ, USA) was placed in the sample and then placed between the DART ion source and the MS inlet. Positive ion MS data were obtained over a *m/z* range of 60–700 and relative percent abundance was obtained for the indigoidine ion. Standards were run after sampling each colony and mass spectral data were monitored in real time to ensure no residual indigoidine remained after each sample. DART-MS is only semi-quantitative due to the potential for differential ionization, suppression of ions, and/or changes in sample concentration in the DART ion source (Sanchez et al., 2011). Therefore, relative indigoidine ion abundance was used to generate heatmaps representing a gradient from less abundance (black) to more abundance (red).

### Measurement of Indigoidine Production by *Leisingera* sp. JC1 in Co-culture

JC1 bacterial inoculum was prepared by adding JC1 colonies into 5 mL of SWT_ng_ media in a 24 deep well plate, incubated for 24 h at room temperature while shaking at 200 rpm. Bacterial inocula for the vibrios were prepared by adding bacterial colonies of each species separately into 5 mL of SWT_ng_ media in 24 deep well plates, incubated for 2 h at 28°C while shaking at 200 rpm. All bacterial inocula (JC1, *V. fischeri*, *V. anguillarum*, *V. parahaemolyticus*) were adjusted to OD_600_ 0.1 prior to use.

Co-cultures of JC1 with individual vibrios were prepared by adding 1 mL of adjusted JC1 inoculum to 10 mL SWT_ng_ media in 125 mL baffled flasks, incubated for 24 h at room temperature while shaking at 125 rpm, followed by addition of 200 μL of *V. fischeri, V. anguillarum*, or *V. parahaemolyticus*. After addition of the vibrio strain, co-cultures were incubated for an additional 24 h at room temperature while shaking at 125 rpm. Monocultures of JC1, *V. fischeri*, *V. anguillarum*, and *V. parahaemolyticus* were prepared by adding 1 mL of adjusted inoculum to 10 mL SWT_ng_ media in 125 mL baffled flasks, incubated for 48 h while shaking at 125 rpm.

All co-cultures and monocultures were extracted using the indigoidine enriched protocol described above. LC–MS data was obtained on the Agilent LC–MS system described above, using an isocratic method to ensure minimal baseline variation (10% acetonitrile in H_2_O with 0.1% formic acid over 15 min at a flow rate of 1 mL/min with 20 μL injection volume). Extracts were prepared at 5 mg/mL in DMSO. Indigoidine was detected and quantitated via measurement of area under the curve at UV absorbance 299 nm and confirmed by MS.

## Results and Discussion

### Genome Characteristics and General Metabolism

#### Taxonomic Placement of JC1

*Leisingera* sp. JC1 has a draft genome size of 5.19 Mb and GC content of 62.3% (**Table 1**), which is average for members of the roseobacter clade and similar to other squid-associated isolates (Collins et al., 2015). This larger genome size reflects the generalist lifestyle and ability to use diverse energy sources common of roseobacters (Newton et al., 2010). The *repABC* genes for plasmid replication are present as well as *tra* genes necessary for conjugative plasmid transfer, indicating the potential presence of extrachromosomal DNA. Further sequencing is necessary to confirm the number, size, and content of these putative plasmids.

**Table 1:** Genome statistics of *Leisingera* sp. JC1.

Genome size (Mb)	Number of contigs	N_50_ (bp)	G + C content (%)	Number of genes	Missing genes^∗^ (% of total)	Fold coverage
5.19	168	123,213	62.3	5,074	54 (1.1)	37

*^∗^As predicted by the RAST server (Aziz et al., 2008).*

**FIGURE 1:**
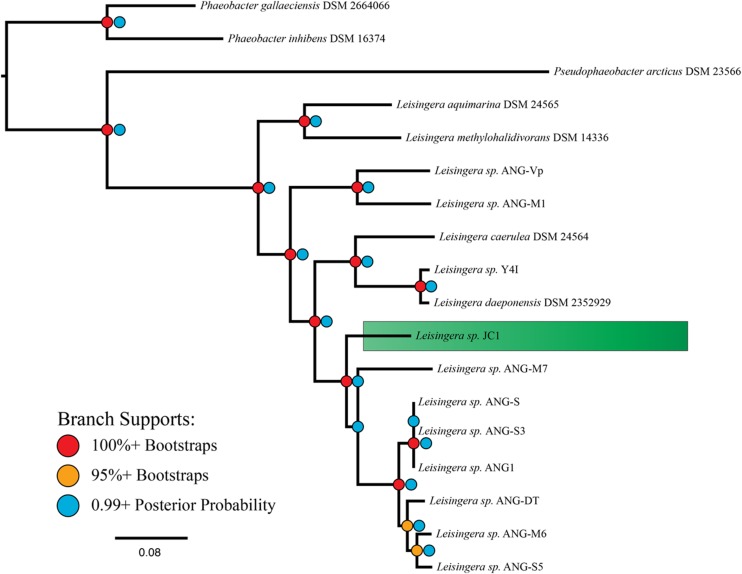
**MLSA analysis places *Leisingera* sp. JC1 in a sister group to other previously isolated ANG bacteria.** Based on a comparison of 33 single-copy housekeeping genes, the egg jelly coat (JC) isolate JC1 is placed in the previously described roseobacter clade, “Clade 1” (Newton et al., 2010), along with seven previously isolated ANG bacteria (Collins et al., 2015). The phylogenetic tree reflects both Bayesian inference and maximum-likelihood methods with both posterior probability and bootstrap supports displayed.

Phylogenetic reconstruction methods (Bayesian and maximum-likelihood) used with the 33-gene concatenation returned identical topologies with overall strong statistical supports (**Figure 1**), placing JC1 close to the *Leisingera* taxa previously isolated from the ANG. Average nucleotide identity (ANI) and *in silico* DNA–DNA hybridization estimates (*is*DDH) support this placement. JC1 had higher ANI (90.5–91.7%) (Supplementary Table S2) and *is*DDH (38.8–44.6%) values with the ANG isolates than with any other *Leisingera* sp. These results also show that JC1 does not group with either *Leisingera* sp. ANG-M7 or the ANG1 group, but is still related to both (**Figure 1**; Supplementary Table S3), which is not unusual since other ANG isolates also fall outside the main ANG1 clade (Collins et al., 2015).

There are indications that JC1 may be more similar to *Leisingera* sp. ANG-M7 than to the *Leisingera* sp. ANG1 group. Both the ANI and *is*DDH values between JC1 and ANG-M7 are elevated in comparison to their values with the ANG1 group. There are no support statistics for ANI so it is uncertain if the 1.2% (JC1-M7 ANI versus JC1 compared to the ANG group) and 1.6% (M7-JC1 ANI versus M7 compared to the ANG group) higher values are significantly different. However, *is*DDH values are supported by 95% confidence intervals. The lower interval for JC1-M7 does not overlap with the upper interval for any comparison with a member of the ANG group, suggesting the *is*DDH values are significantly different. Additionally, the Bayesian inference found a small fraction of topologies in which the placements of JC1 and ANG-M7 were reversed, while the maximum-likelihood analysis found this occurrence in 33 of 100 bootstrap replicates. Overall, these analyses suggest that *Leisingera* sp. JC1 is distinct from, but related to the current ANG isolates.

Isolates having similar pigmentation to *Leisingera* sp. JC1 were cultured from other egg clutches, an ANG, and ovary from different females (data not shown). Among these, colonies with a similar dark blue morphology were isolated from the JCs of 1 and 23 day old eggs laid by different females. Similar colonies were isolated from the ANG of one of these females and the ovary of another female. Preliminary 16S sequencing placed two of these isolates in the genus *Leisingera* (data not shown), and further sequencing will reveal if these are the same strain as JC1. In addition, the production of the pigment indigoidine was confirmed by these additional strains (see below). These data suggest that *Leisingera* sp. JC1 and/or other indigoidine-producing strains may be selected for in the ANG/JC symbiosis.

#### Primary Metabolism

*Leisingera* sp. JC1 has a complete Entner-Duodoroff pathway and tricarboxylic acid cycle for metabolism of glucose. JC1 lacks any orthologs of phosphofructokinase, a major enzyme of glycolysis, but does contain a glucokinase and two distinct glucose-6-phosphate-1-dehydrogenases (GAPDHs). A glucose-6-phosphate-1-dehydrogenase (GPDH) is present, which catalyzes the first step of the alternative pathways for glucose metabolism, indicating that the Entner-Duodoroff pathway is probably used instead of glycolysis. *Leisingera* sp. JC1 only has the first two enzymes of the oxidative pentose phosphate pathway, but any 6-phosphate-gluconate produced can be further dehydrated by the Entner-Duodoroff pathway. Glycolate is a dissolved organic carbon often excreted by phytoplankton, and can be a carbon source for marine heterotrophic bacteria (Edenborn and Litchfield, 1985). *Leisingera* sp. JC1 is predicted to oxidize glycolate to glyoxylate by a glycolate oxidase. JC1 has one system for glycerol uptake, the Ugp system, which can transport glycerol-3-phosphate against the concentration gradient. Sulfur oxidation genes are present, as well as a complete denitrification pathway with a copper-containing nitrite reductase. An assimilatory nitrate reductase is also present, which can convert nitrate to nitrite. An ammonia assimilation pathway is present with a ferrodoxin-dependent GOGAT, but no adenylyltransferase gene (GlnE) is present.

#### Transport

The high-affinity inorganic phosphate transport genes *pstABCS* and their regulatory genes *phoBUR* are present in JC1. The siderophore biosynthesis genes *asbAB* and *siderX456*, which encode high-affinity iron chelators, and the ferric iron ABC transporter, *pitADC*, are also present. JC1 has ABC transporters for dipeptides, oligopeptides, branched-chain amino acids, alkylphosphonate, and tungstate. The tripartite ATP-independent periplasmic (TRAP) transporter genes *dctMPQ* are present for unknown substrates, as well as the twin-arginine translocation (TAT) system genes, *tatABC*.

*Leisingera* sp. JC1 contains all 13 genes that encode the structural proteins essential for the Type VI Secretion System (T6SS) to function (Cianfanelli et al., 2016). The T6SS is a one-step mechanism for delivery of effectors across the Gram-negative outer membrane and membrane of the target cell, be it bacterial or eukaryotic. Widespread amongst the *Proteobacteria*, some T6SSs have been implicated in eukaryotic virulence (Pukatzki et al., 2006; Sana et al., 2012), but the majority are believed to play a role in bacterial competition (Hood et al., 2010; Schwarz et al., 2010). While it is possible for one T6SS system to affect both bacterial and eukaryotic targets (Jiang et al., 2014) it is believed that the system evolved for interactions with other bacteria, even in the case of intraspecific competition (Unterweger et al., 2014). Little work has been done, however, to investigate the role of T6SSs in beneficial host-symbiont relationships. Eleven of the 12 previously described ANG isolates also possess a T6SS (Collins et al., 2015), and it is possible that this system plays a role in interactions with other ANG or JC bacteria and/or the squid host. In the ANG, bacteria are partitioned into densely packed, epithelium-lined tubules, where each tubule is dominated by a particular taxon (Collins et al., 2012). These ANG/JC isolates may utilize the T6SS to outcompete other bacteria to establish colonization of a single tubule. While *Leisingera* sp. JC1 groups closely with other ANG isolates that also possess a T6SS (**Figure 1**, Collins et al., 2015), intraspecific effectors may facilitate competition between these strains, since ANG tubules are often highly pigmented with a single color (e.g., all dark blue matching the pigmentation of JC1 or all red-orange matching the pigmentation of several ANG isolates). Future studies will investigate the nature of JC1’s T6SS effector proteins in the ANG symbiosis. There are numerous classes of evolved effector VgrG proteins, each with their own enzymatic function (reviewed in Durand et al., 2014). Understanding the number and type of effectors that JC1 can produce and deliver may help elucidate any role in the symbiosis.

### Secondary Metabolite Biosynthesis

Analysis with the antibiotic and Secondary Metabolite Analysis Shell (antiSMASH, Weber et al., 2015) predicted several potential secondary metabolite biosynthesis gene clusters (Supplementary Table S4). These results included three separate siderophore clusters, one bacteriocin, one HSL, one type 1 polyketide synthase (T1 PKS), one other PKS (not type 1,2,3, or *trans*-AT), and two clusters classified as “other.” Of these two “other” clusters, one contains the biosynthesis cluster for the known antimicrobial metabolite, indigoidine (Cude et al., 2012), while the other contains a previously described putative hybrid polyketide synthase/non-ribosomal peptide synthetase (PKS/NRPS) gene cluster known to be conserved amongst roseobacters (Martens et al., 2007). This PKS/NRPS gene cluster encodes a polyketide synthase, glycosyl transferase, non-ribosomal peptide synthetase, and phosphopantetheinyl transferase, but the product of this cluster has not yet been identified. The top homologous gene cluster of the T1 PKS is 45% similar to a cluster in the ANG isolate, *Leisingera* sp. ANG-M7. While some roseobacters are capable of producing the novel secondary metabolite TDA (Bruhn et al., 2006, 2007; Geng et al., 2008), genes for synthesis of this molecule were not found nor was the molecule detected via LC–MS (data not shown).

### Quorum Sensing

**FIGURE 2:**
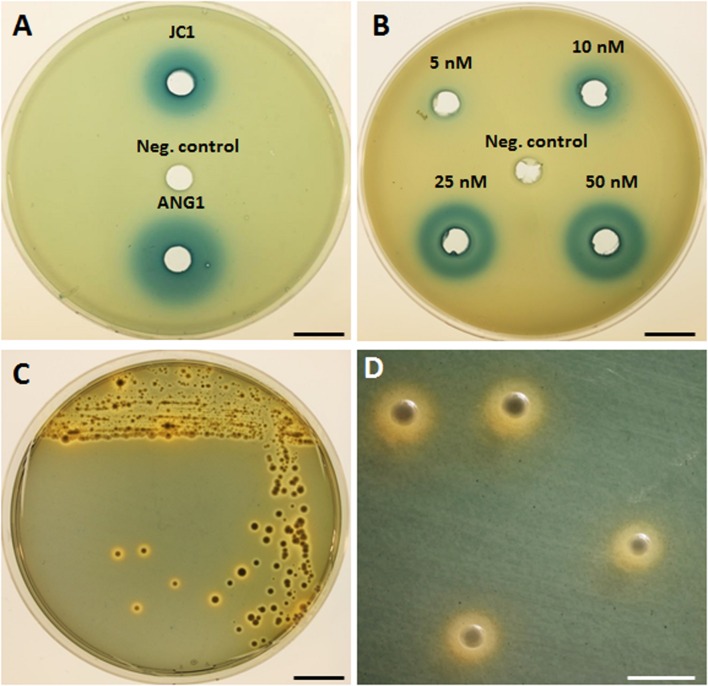
**Detection of homoserine lactone production and siderophore production by *Leisingera* sp. JC1. (A)** Homoserine lactones were detected by β-galactosidase activity in cell-free supernatant of *Leisingera* sp. JC1 and compared with previously tested *Leisingera* sp. ANG1 (Collins et al., 2015); SWT broth was used as a negative control. **(B)** Dilution of *N*-3-oxohexanoyl homoserine lactone used as a positive control for the HSL assay; DMSO was the HSL standard solvent and was the negative control. **(C)** *Leisingera* sp. JC1 plated on CAS agar. Sequestration of iron changes the media from blue to orange, indicating siderophore production. **(D)** Magnified view of JC1 colonies from the plate in **(C)**, showing the orange halos in the media indicative of siderophore production. Scale bars, 1.5 cm **(A–C)**, 4 mm **(D)**.

AntiSMASH predicted one *luxIR* homolog in *Leisingera* sp. JC1, flanked by an acyltransferase, crotonyl-CoA reductase, helicase, and oxidoreductase, similar to the previously published gene arrangement in bacterial isolates from the ANG (Collins et al., 2015). Production of HSLs by JC1 was confirmed in the *A. tumefaciens* NTL4 reporter assay, in which cell-free supernatant of a JC1 culture did induce β-galactosidase activity, indicating the presence of HSLs (**Figures 2A,B**). When compared to a dilution series of the *N*-3-oxohexanoyl HSL, JC1 produced a halo similar to that seen by 25 nM of HSL standard. The HSL production of JC1 was also slightly less than that of a closely related ANG isolate, *Leisingera* sp. ANG1.

Understanding the gene regulation by quorum sensing will be an important avenue of research for *Leisingera* sp. JC1 and the other *E. scolopes* ANG isolates due to the different habitats these bacteria experience. It is hypothesized that cephalopod ANGs are colonized via horizontal transmission from the environment (Kaufman et al., 1998), and potential symbionts must switch from living at very low cell densities in the seawater to very high cell densities in the ANG tubules (Collins et al., 2012). When ANG bacteria are deposited into the JC layers of eggs, these bacteria again experience a switch from the very high densities of the ANG to a lower density in the eggs. Due to this change in environments and cell densities, quorum sensing may play a role in gene regulation for ANG/egg JC bacteria.

Quorum sensing is also important in host-microbe interactions involving other roseobacters. For example, quorum sensing regulates motility and biofilm formation during host colonization in the sponge symbiont *Ruegeria* sp. KLH11 (Zan et al., 2012) and is necessary for colonization of the alga, *Ulva australis* by *Phaeobacter gallaeciensis* 2.10 (Rao et al., 2007). In other roseobacters, quorum sensing regulates secondary metabolite production, such as TDA in *Phaeobacter gallaeciensis* (Berger et al., 2011). In the indigoidine producing roseobacter, *Leisingera* sp. Y4I, there are two quorum sensing systems that regulate indigoidine production, *pgaIR* and *phaIR* (Cude et al., 2015). The JC1 *luxI* homolog has a 72% amino acid similarity to *pgaI* (RBY4I_1689) in Y4I, and the JC1 *luxR* homolog has an 81% amino acid similarity to *pgaR* (RBY4I_3631) in Y4I. The second set of *luxIR* homologs in Y4I, *phaIR* (RBY4I_3464 and RBY4I_1027), is not present in JC1. PgaI synthesizes the C8-HSL, produced by several proteobacteria, while PhaI synthesizes the 3OHC_12:1_-HSL, which may be species specific. JC1 lacking the *phaIR* system may reflect its divergence from *Leisingera* sp. Y4I. Further analyses will be needed to understand if indigoidine production in *Leisingera* sp. JC1 is regulated by quorum sensing.

### Siderophore Production

Three separate siderophore biosynthesis gene clusters were detected in the genome, as described above, and production of iron chelators was confirmed by plating on CAS agar (**Figures 2C,D**). Appearance of an orange halo around colonies indicates that iron was sequestered from the chrome-azurol S dye in the media. Siderophores are high-affinity iron chelators, and can provide a growth advantage to cells in iron-limited environments, such as in seawater and in colonization of hosts. Although, the presence of siderophore biosynthesis genes in the genomes of currently sequenced roseobacter clade members is rare, 10 of the 12 previously sequenced *E. scolopes* ANG roseobacter symbionts did have the genes and/or demonstrate production of siderophores (Collins et al., 2015). Similar to the majority of the squid-associated roseobacter clade, the *Leisingera* sp. JC1 genome contains siderophore biosynthesis genes, indicating that siderophore production may play a role in the ANG symbiosis.

### Indigoidine Biosynthesis Genes

**FIGURE 3:**
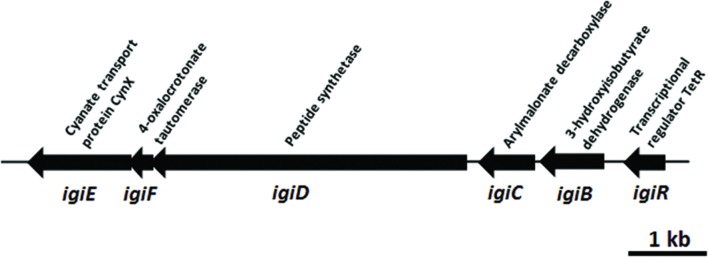
***Leisingera* sp. JC1 indigoidine biosynthesis operon.** Operon organization obtained from the published genome sequence (OBY26161.1, OBY26149.1, OBY26162.1, OBY26150.1, OBY26151.1, OBY26152.1). Arrows drawn to scale.

The indigoidine biosynthesis gene cluster in *Leisingera* sp. JC1 contains all six biosynthesis genes previously described for *Leisingera* sp. Y4I (Cude et al., 2012) and shares a similar genome arrangement (**Figure 3**). To confirm the presence of individual members of the indigoidine biosynthesis gene cluster, primers were designed to three different components of the pathway, the non-ribosomal peptide synthetase (*igiD*), the transcriptional regulator (*igiR*), and one of the three indigoidine modification genes (*igiC*). The presence of these genes in JC1 genomic DNA was confirmed by PCR (Supplementary Figure S1).

Indigoidine biosynthesis genes have been detected in a diverse group of bacteria, including the *Actinobacteria* (*Streptomyces)*, and *Alpha-, Beta-*, and *Gamma-proteobacteria*. The JC1 indigoidine biosynthesis operon shares the closest homology to the operon in *Leisingera* sp. Y4I, with 90–95% amino acid similarity for all gene products (**Table 2**). Other indigoidine biosynthesis operons share the non-ribosomal peptide synthetase, *igiD*, but many lack the same accessory genes required to modify indigoidine. When compared to other indigoidine producing strains, the *igiD* of JC1 is functionally homologous to other NRPS genes, sharing 49–53% amino acid similarity with *Vogesella indigofera*, *Streptomyces lavendulae*, and *Dickeya dadantii* 3937 (**Table 2**). A comparison with the genome of *Leisingera* sp. Y4I also confirmed that the indigoidine gene cluster is shared between these strains although absent from related ANG isolate *Leisingera* sp. M7 (Supplementary Figures S4 and S5).

**Table 2:** Comparison of indigoidine biosynthesis operon in *Leisingera* sp. JC1 to other indigoidine producing strains.

Gene	Annotation	% Amino acid identity to *Leisingera* sp. Y4I operon	% Amino acid identity to *Vogesella indigofera* operon	% Amino acid identity to *Streptomyces lavendulae* operon	% Amino acid identity to *Dickeya dadantii* 3937 operon
*igiE*	Cyanate transport protein, CynX	95	58	NA	NA
*igiF*	4-oxalocrotonate tautomerase	91	NA^∗^	NA	NA
*igiD*	Peptide synthetase	91	53	50	49
*igiC*	Arylmalonate decarboxylase	95	56	NA	NA
*igiB*	Hydroxyisobutyrate dehydrogenase	93	51	NA	NA
*igiR*	Transcriptional regulator, TetR	90	42	NA	NA

*^∗^NA, not applicable, no homolog of the gene present in that organism.*

### Detection of Indigoidine Production by *Leisingera* sp. JC1

**FIGURE 4:**
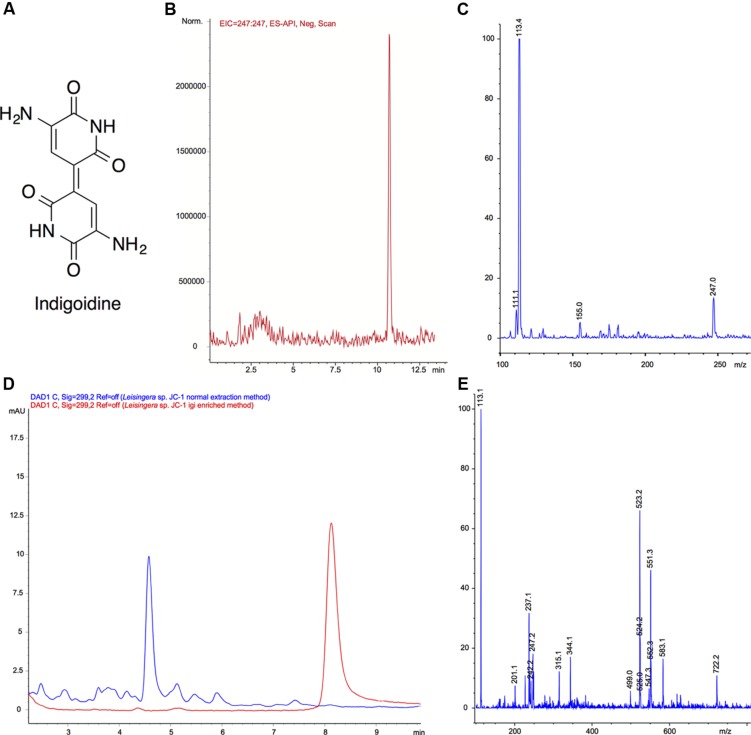
**Mass spectral confirmation of indigoidine production by *Leisingera* sp. JC1 and comparison of normal and igi enriched extracts. (A)** Structure of indigoidine. **(B)** Extracted ion chromatogram (EIC) of indigoidine to confirm presence in extract ([M-H]^-^ 247.0) using a previously reported LC–MS solvent gradient (Yu et al., 2013). **(C)** Negative ionization mass spectrum of peak at 10.7 min. The [M-H]^-^ peak of 247.0 is consistent with a molecular formula of C_10_H_7_N_4_O_4_, confirming the presence of indigoidine (248.19 g/mol). **(D)** LC–MS UV chromatogram overlays (299 nm) of normal extract (blue) with indigoidine (igi) enriched extract (red). The peak eluting at t_R_ 8.2 min in the igi enriched extract was confirmed to be indigoidine with a purity of 91.1% (t_R_ different due to use of isocratic solvent system to minimize baseline variation). Although, a small peak was present at t_R_ 8.2 min in the normal extract, this integrates to only 0.8% indicating that the *Leisingera* sp. JC1 normal extract contains negligible amounts of indigoidine. **(E)** Negative ionization mass spectrum of peak at t_R_ 8.2 min for normal extraction method. Although there was an ion corresponding to [M-H]^-^ of 247.2, this was not the most dominant ion and the more quantitative UV analysis in D shows only a very small peak, indicative of negligible indigoidine in the JC1 normal extract.

Because of the distinctive morphology and the genetic evidence for indigoidine biosynthesis, *Leisingera* sp. JC1 was cultured and extracted to obtain chemical evidence of indigoidine production. Using a three-step culture process, a deep blue liquid culture was obtained. However, upon extraction using a typical resin-based organic extraction protocol, most of the blue color was insoluble in organic solvents and little evidence of indigoidine production was observed via liquid chromatography-mass spectrometry (LC–MS, see **Figures 4D,E**), integrating to only 0.8% of the JC1 normal extract and indicative of negligible indigoidine extraction using this method. Therefore, an indigoidine enriched extraction protocol was utilized to pellet the insoluble indigoidine away from other media and cellular components followed by dissolving the sample in DMSO (Yu et al., 2013), resulting in an indigoidine enriched extract with 91.1% indigoidine. Analysis via LC–MS confirmed the presence of indigoidine (**Figure 4A**) in the indigoidine enriched extract (**Figures 4B,C**) with a peak eluting at 10.7 min with an [M-H]^-^ of 247.0, consistent with the molecular weight and fragmentation pattern of indigoidine (248.2 g/mol) and in agreement with literature precedent (Yu et al., 2013). In addition, indigoidine was detected in two other JC and ANG isolates that exhibited a similar dark blue coloration in culture (data not shown).

### Antibacterial Activity of *Leisingera* sp. JC1

#### Zone of Inhibition

**FIGURE 5:**
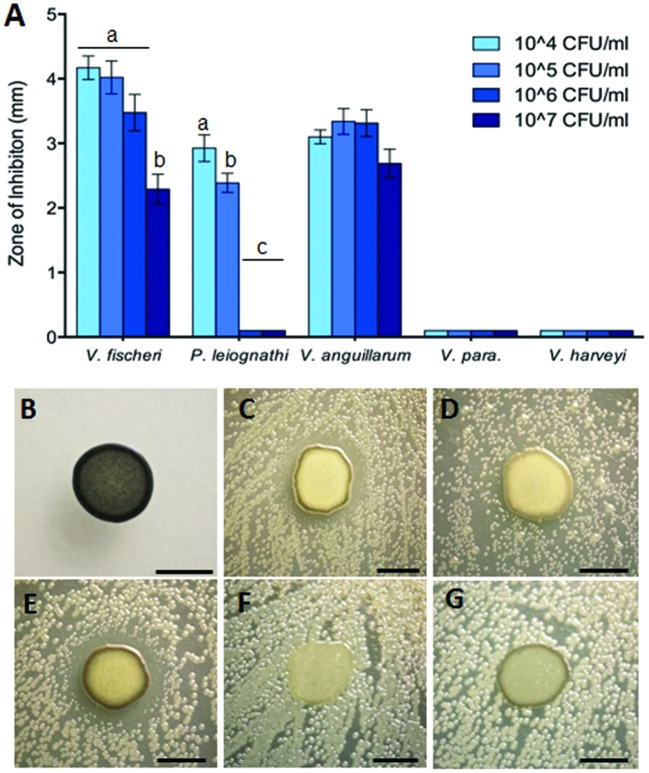
***Leisingera* sp. JC1 differentially inhibits pathogenic and non-pathogenic vibrio species. (A)** Zones of inhibition around JC1 colonies were measured with increasing lawn densities of each vibrio species. *Leisingera* sp. JC1 differentially inhibited five vibrio strains. Significantly greater inhibition of *Vibrio fischeri* was seen between the 10^4^–10^6^ CFU/ml and the 10^7^ CFU/ml lawn density. Significantly greater inhibition of *Photobacterium leiognathi* was seen between the 10^4^ and 10^5^ CFU/ml lawn densities, as well as between the 10^4^–10^5^ and the 10^6^–10^7^ CFU/ml densities. JC1 inhibited *Vibrio anguillarum* uniformly across all lawn densities and did not inhibit *Vibrio parahaemolyticus* or *Vibrio harveyi*. Letters indicate significantly different groups based on results from *post hoc* Tukey tests (see Results and Discussion). Representative 24 h images of **(B)** JC1 plated alone on YTSS agar and with 10^6^ CFU/ml of **(C)** *V. fischeri*, **(D)** *P. leiognathi*, **(E)** *V. anguillarum*, **(F)** *V. parahaemolyticus*, and **(G)** *V. harveyi*. Scale bars: 5 mm **(B–G)**.

Zone of inhibition assays were performed to test the ability of *Leisingera* sp. JC1 to inhibit other marine bacteria, both free-living and symbiotic (Supplementary Table S3). JC1 was tested against the *E. scolopes* light organ symbiont, *V. fischeri* ES114; another bioluminescent member of the *Vibrionaceae*, *P. leiognathi* KNH6, isolated from Hawaiian seawater; *V. harveyi* B392; *V. parahaemolyticus* KNH1 and *V. anguillarum* 775. These bacteria were plated at lawn densities from 10^4^ to 10^7^ CFU/mL to test the efficacy of possible inhibition at varying densities which more closely reflect biologically relevant concentrations. Overall, *Leisingera* sp. JC1 differentially inhibited the five vibrios tested (**Figure 5**). For two of the strains tested, *V. fischeri* (*F*_3,76_ = 12.63, *P* < 0.0001) and *P. leiognathi* (*F*_3,60_ = 137.5, *P* < 0.0001), JC1 showed significantly greater inhibition at lower lawn densities (**Figure 5A**; Supplementary Figure S2). When measured ZOIs were normalized for variations in JC1 colony diameter, there was an average 4.2 mm ZOI at 10^4^ CFU/mL of *V. fischeri*, while at the 10^7^ CFU/mL density, there was a 2.3 mm ZOI (Supplementary Figures S2I–L). A multiple comparisons *post hoc* Tukey test determined that the ZOI for the 10^4^–10^6^ CFU/mL lawn densities of *V. fischeri* were significantly greater than the ZOI at the 10^7^ CFU/mL density. The change in ZOI with test strain lawn density was most apparent for *P. leiognathi*, where the average ZOI at the 10^4^–10^5^ CFU/mL lawn densities ranged from 2.4 to 2.9 mm, and then dropped to 0 mm at the 10^6^–10^7^ CFU/mL densities (**Figures 5A,D**; Supplementary Figures S2A–D). A multiple comparisons *post hoc* Tukey test showed that the ZOI at 10^4^ CFU/mL of *P. leiognathi* was significantly different from the ZOI at 10^5^ CFU/mL, and that both ZOIs at 10^4^ and 10^5^ CFU/mL were significantly different from the 10^6^–10^7^ CFU/mL results. *Leisingera* sp. JC1 showed a trend toward inhibition of *V. anguillarum* with ZOIs ranging from 2.7 to 3.3 mm (**Figures 5A,E**; Supplementary Figures S2E–H) although, a one-way ANOVA determined that the ZOIs were not statistically different (*F*_3,60_ = 2.553, *P* = 0.0639). No inhibition was observed when JC1 was tested against *V. parahaemolyticus* or *V. harveyi* at any lawn density (**Figures 5A,F,G**; Supplementary Figures S2M–P).

*Leisingera* sp. JC1 was also tested in a ZOI assay against the 12 previously described ANG isolates (Collins et al., 2015) and one additional ANG isolate, *Muricauda* sp. ANG21. All ANG isolates were only tested at a lawn density of approximately 10^8^ CFU/mL. Inhibition was observed against *Ruegeria* sp. ANG-S4, with an average ZOI of 6.3 mm (±0.7) and against *Muricauda* sp. ANG21, with an average ZOI of 5.9 mm (±0.6; Supplementary Figure S3). *Leisingera* sp. JC1 was not able to inhibit any of the other *Leisingera* spp. previously isolated from ANGs. Since partitioning between bacterial taxa is observed in the ANG tubules some activity against other ANG isolates may contribute to competition between strains during colonization (Collins et al., 2012).

#### 96-Well Liquid Assay

**FIGURE 6:**
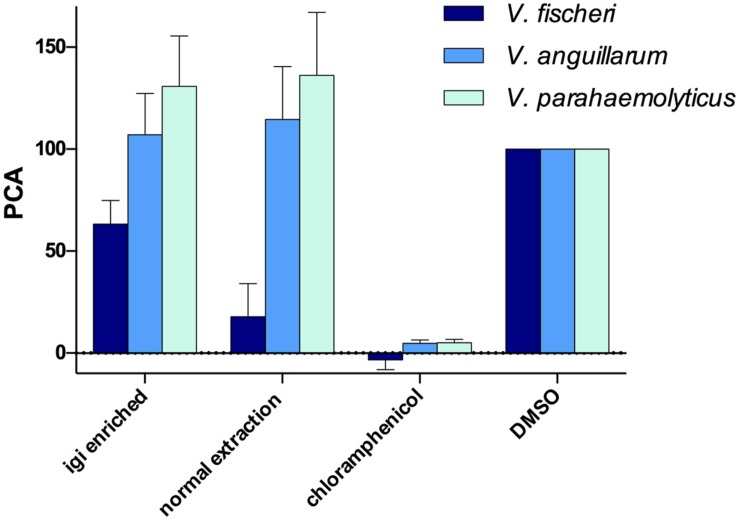
***Leisingera* sp. JC1 extracts inhibit *V. fischeri* in 96-well liquid assays.** JC1 cultures were grown at large scale and extracts were prepared using either an indigoidine enriched procedure (igi enriched) or a normal extraction method. All extracts were screened at 500 μg/mL against *V. fischeri*, *V. anguillarum*, and *V. parahaemolyticus*. The JC1 normal extract strongly inhibited growth of *V. fischeri* (PCA 17.9 ± 9.3) with less activity of the JC1 igi enriched extract against *V. fischeri* (PCA 63.3 ± 6.7). No inhibition of *V. anguillarum* or *V. parahaemolyticus* was observed. Data shown are average PCA values from three experiments (PCA, percent control activity, as compared with DMSO negative control).

Both the normal and indigoidine enriched JC1 extracts were screened for activity using 96-well plate liquid assays with several of the vibrios tested above, including *V. fischeri* ES114, *V. anguillarum* 775, and *V. parahaemolyticus* KNH1 (**Figure 6**). Both extracts were initially tested at 500 μg/mL with minimum inhibitory concentrations (MICs) determined for active samples. The JC1 normal extract was found to strongly inhibit growth of *V. fischeri* with a PCA value of 17.9 ± 9.3 during screening and was determined to have a MIC of 250 μg/mL. The JC1 indigoidine enriched extract also exhibited moderate inhibition of *V. fischeri* with a PCA of 63.3 ± 6.7.

In contrast to the ZOI data above, no inhibition was observed for either extract when tested against *V. anguillarum*, potentially due to differences between the activity of indigoidine in agar versus liquid assays, as seen with *Leisingera* sp. Y41 and hypothesized to result from changes in the redox state of indigoidine (Cude et al., 2012). These results may also be attributed to differences in the chemical composition between extracts and the bacteria *in situ* (e.g., aqueous soluble metabolites are generally excluded from the extraction protocols used in this study). Neither JC1 extract inhibited *V. parahaemolyticus*, in agreement with the ZOI data above.

Previous studies with a mutant of *Leisingera* sp. Y4I that did not produce indigoidine suggested that production of the compound is required for inhibition of *V. fischeri* (Cude et al., 2012). However, with the more potent inhibition of *V. fischeri* seen in the JC1 normal extract versus the indigoidine enriched extract in this study (**Figure 6**), indigoidine production does not seem to be the only mechanism of inhibition for *Leisingera* sp. JC1. Given that the JC1 normal extract contains only minimal amounts of indigoidine (0.8% as discussed above), the bacterium may be utilizing other secondary metabolites in conjunction with indigoidine for chemical defense. The JC1 genome includes several other secondary metabolite biosynthetic gene clusters for HSL, siderophore, bacteriocin, PKS, and PKS/NRPS production and thus *Leisingera* sp. JC1 likely utilizes one or more of the compounds encoded by these pathways for chemical defense, in addition to the defensive capabilities attributed to indigoidine. Creating an indigoidine mutant of *Leisingera* sp. JC1 will help test this hypothesis, in conjunction with identification of additional metabolite(s) responsible for JC1 antimicrobial activity.

### Localization of Indigoidine Production by *Leisingera* sp. JC1

**FIGURE 7:**
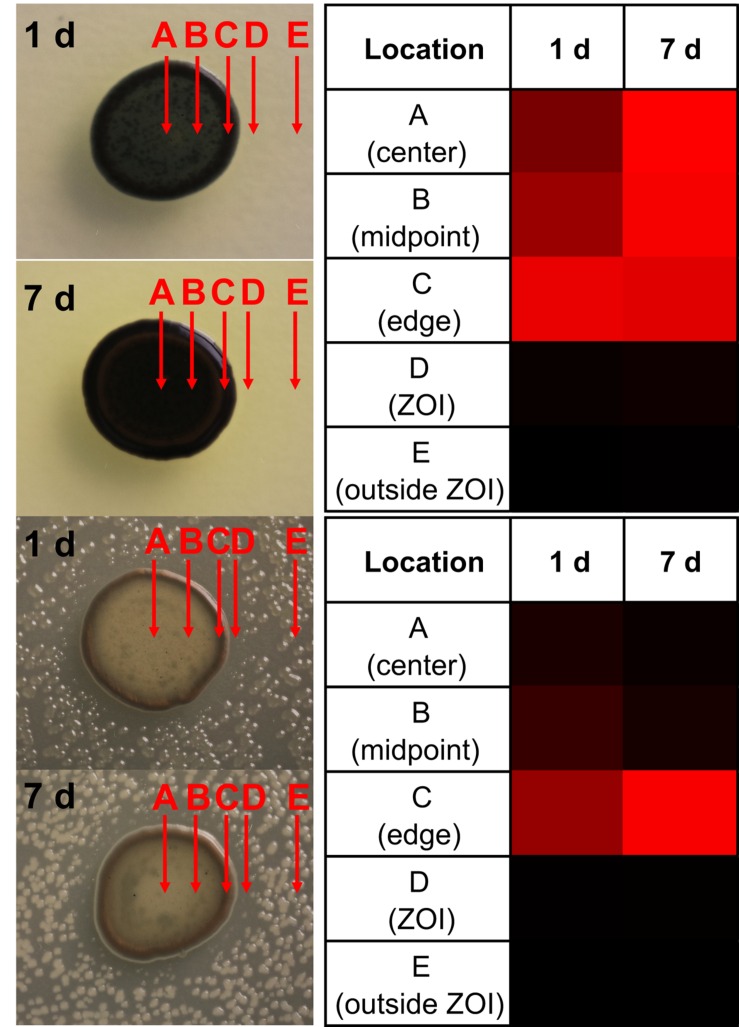
***Leisingera* sp. JC1 localizes indigoidine production to outer edge of colony in the presence of *V. fischeri*.** Five locations (A–E) were analyzed for the presence of indigoidine via direct analysis in real time-mass spectrometry (DART-MS) over 7 days. (A) In the absence of *V. fischeri*, indigoidine production was more uniformly distributed across the colony. (B) In the presence of *V. fischeri*, indigoidine production was primarily localized to outer edge of colony (location C) whereas minimal indigoidine was detected in the center of the colony (location A). Very little indigoidine was detected in the ZOI or outside ZOI in the presence or absence of *V. fischeri*. Heatmaps represent a gradient of indigoidine ion abundance from less abundance (black, 0%) to more abundance (red, 100%).

While performing ZOI assays, there was a dramatic change in colony pigmentation of *Leisingera* sp. JC1 when grown alone (**Figure 5B**) as compared to growth under challenge with various vibrio strains (**Figures 5C–G**). Deep blue pigment production was observed uniformly when JC1 was grown in monoculture and appeared to localize to the outer edges of the colonies when presented with vibrio strains. Direct analysis in real time-mass spectrometry (DART-MS) is an ambient ionization technique in which samples can be analyzed without sample preparation or extraction (Sanchez et al., 2011). DART-MS was utilized to chemically confirm the visual observations of localization of indigoidine production of JC1 in monoculture and co-culture with *V. fischeri* over the course of 7 days (**Figure 7**). Five locations were selected on each colony including center (A), midpoint (B), edge (C), ZOI (D), and outside ZOI (E). In the absence of *V. fischeri*, indigoidine was uniformly produced throughout JC1 colonies (locations A–C). However, in the presence of *V. fischeri* there was little to no indigoidine production in the center or midpoints of JC1 colonies, but intense indigoidine detected around colony edges (**Figure 7**, location C only). Indigoidine was only minimally detected in the ZOI or outside the ZOI for either monoculture or co-culture. Trends in the localization of indigoidine production were even more apparent upon measurement after 7 days.

There are several examples of pigment production being induced when in the presence of other bacteria, such as in *Staphylococcus aureus* when co-cultured with *Pseudomonas aeruginosa* (Antonic et al., 2013) or production of a red pigment by *Streptomyces lividans* TK23 when co-cultured with *Tsukamurella pulmonis* TP-B0596 (Onaka et al., 2011). Pigment production can also be induced under other stress response conditions, such as protection from UV radiation (Tong and Lighthart, 1997). Pigment production has also been tied to photosynthesis (Orf and Blankenship, 2013), however, *Leisingera* sp. JC1 lacks genes associated with photosynthesis or carbon fixation (data not shown).

When grown alone, *Leisingera* sp. JC1 exhibited a uniform blue–black pigmentation across the colony which was confirmed by mass spectrometry to be essentially uniform production of indigoidine. Secondary metabolite biosynthesis is an energy intensive endeavor and production of antimicrobial compounds would typically be thought to be reserved for defensive situations. Since indigoidine is produced throughout the colony when in monoculture, and given its relatively moderate antibacterial activity as suggested by assays with the indigoidine enriched extract, it is also possible that indigoidine serves multiple functions for *Leisingera* sp. JC1. However, *Leisingera* sp. JC1 localized indigoidine production to the outer edges of the colony when co-cultured with *V. fischeri* and other vibrios. If utilized as a defensive compound, indigoidine may be localized to points of direct interaction with other microorganisms. Secondary metabolite production can be localized to susceptible locations such as in plants, sponges, and other sessile terrestrial and marine organisms (Amsler et al., 2001; Furrow et al., 2003; Van Dyck et al., 2010). The role of *Leisingera* sp. JC1 has yet to be examined directly in the ANG symbiosis but localized production of indigoidine or other secondary metabolites may play a role in egg defense or inhibition of other bacteria from colonizing the ANG (see conclusions below).

### Regulation of Indigoidine Production by *Leisingera* sp. JC1

**FIGURE 8:**
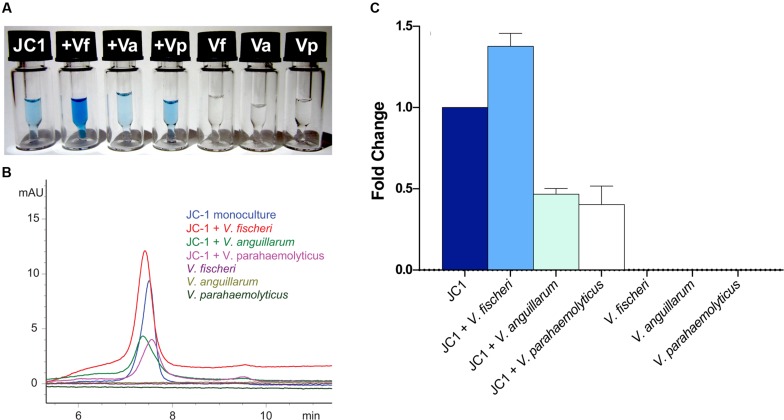
***Leisingera* sp. JC1 upregulates indigoidine production in the presence of *V. fischeri*.** JC1 was cultured alone and in the presence of *V. fischeri*, *V. anguillarum*, and *V. parahaemolyticus* (each vibrio was also monocultured). **(A)** Extracts were prepared in DMSO at 5 mg/mL for LC–MS (from left to right): JC1 monoculture, JC1 + *V. fischeri*, JC1 + *V. anguillarum*, JC1 + *V. parahaemolyticus*, *V. fischeri* monoculture, *V. anguillarum* monoculture, and *V. parahaemolyticus* monoculture. As shown, the highest pigment production was observed when JC1 was co-cultured with *V. fischeri*. **(B)** LC–MS UV chromatograms (299 nm) for all extracts. JC1 indigoidine production was upregulated when co-cultured with *V. fischeri* (red). Down regulation was observed when co-cultured with *V. anguillarum* or *V. parahaemolyticus* (green and pink, respectively). **(C)** Following extraction, indigoidine production was measured using HPLC and quantitated via measurement of the area under the curve for UV absorption at 299 nm. Fold change was calculated as compared with JC1 monoculture.

After observing localized production of indigoidine when grown on solid media with *V. fischeri*, additional co-culture experiments were undertaken in liquid media using several of the vibrios from the antimicrobial assays above. *Leisingera* sp. JC1 was grown in monoculture and in the presence of *V. fischeri*, *V. anguillarum*, and *V. parahaemolyticus*, followed by extraction and measurement of indigoidine production (**Figure 8**). Monocultures of all three vibrios were also grown and extracted as controls. Addition of *V. fischeri* to established cultures of *Leisingera* sp. JC1 resulted in a 1.38 fold increase in indigoidine. Co-cultures with *V. anguillarum* and *V. parahaemolyticus* resulted in a decrease in indigoidine production of approximately 0.5 fold for both organisms. Vibrio monocultures confirmed that these species do not produce indigoidine. Changes in indigoidine production were also visually evident with darker, more intense blue observed for extracts cultured with *V. fischeri* in comparison with JC1 monoculture, as well as lighter blue extracts observed for *V. anguillarum* and *V. parahaemolyticus* co-cultures (**Figure 8A**).

The increase in indigoidine production of JC1 with *V. fischeri* is consistent with the antibacterial activity observed for *Leisingera* sp. JC1 on both solid and liquid media (**Figures 5** and **6**), strengthening the hypothesis that indigoidine may play a protective role in association with *E. scolopes*. In addition, the downregulation of production with *V. anguillarum* and *V. parahaemolyticus* also supports the liquid culture bioassay data (**Figure 6**). The differential antimicrobial activity and indigoidine production between the three vibrios may be due to the purported role of the ANG and JC bacteria in the host. The ability of *Leisingera* sp. JC1 to inhibit *V. fischeri* may be related to the fact that the ANG is located directly posterior to the light organ, which harbors high densities of the sole symbiont, *V. fischeri* (McFall-Ngai, 2014). Each day 95% of viable *V. fischeri* cells in the light organ are expelled directly into the mantle cavity of the host as part of the regulatory mechanisms of that association (Boettcher et al., 1996; Nyholm and McFall-Ngai, 1998). A study from another squid, *Doryteuthis pealeii* (Kaufman et al., 1998) suggests that ANG bacteria are environmentally transmitted during development. Given that *V. fischeri* is not detected in the ANG (Collins et al., 2012), the inhibitory effect of *Leisingera* sp. JC1 and other ANG isolates may prevent *V. fischeri* and other vibrios from colonizing the ANG and thus help shape the consortium during development. Alternatively, inhibition against vibrios may play a role in egg defense since eggs are exposed to seawater for approximately three weeks and vibrios are known to be common members of the bacterioplankton.

## Conclusions

Genome analyses confirm that *Leisingera* sp. JC1 is part of the squid-associated roseobacter clade. Both *in silico* and *in vitro* analyses confirmed the secondary metabolite potential and production of siderophores, acyl-homoserine lactones associated with quorum sensing, and the pigment indigoidine. *Leisingera* sp. JC1 and its extracts had inhibitory activity against a variety of marine bacteria including the light organ symbiont *V. fischeri*. Furthermore, JC1 challenged with *V. fischeri* led to increased localized production of indigoidine as well as an increased production of indigoidine when co-cultured in liquid media. Taken together these results suggest that *Leisingera* sp. JC1 may play a protective role in egg defense and/or in shaping the microbial community of the ANG. The importance of defensive symbioses in nature is becoming increasingly more evident (Flórez et al., 2015). A number of both terrestrial and marine organisms use novel secondary metabolites produced by bacteria toward defense from potential pathogens and fouling microorganisms. Since roseobacters have been found in the ANGs of a number of cephalopods from diverse marine environments (Kaufman et al., 1998; Grigioni et al., 2000; Barbieri et al., 2001; Pichon et al., 2005; Collins et al., 2012) there may be a conserved function of this group in this symbiosis. Further studies from this group may reveal novel compounds that are important for the biology of these associations and that exhibit antimicrobial activity.

## Author Contributions

MB, SN, SG, and AS conceptualized and designed research; SG, AS, MF, and JLG conducted experiments; MB, SN, SG, AS, MF, and JPG analyzed data and wrote the paper.

## Conflict of Interest Statement

The authors declare that the research was conducted in the absence of any commercial or financial relationships that could be construed as a potential conflict of interest.

## Footnotes

1 http://rast.nmpdr.org

2 http://antismash.secondarymetabolites.org
