# Supplementary material for: Leisingera sp. JC1, a Bacterial Isolate from Hawaiian Bobtail Squid Eggs, Produces Indigoidine and Differentially Inhibits Vibrios
Source: Front Microbiol. 2016 Sep 8;7:1342. doi: 10.3389/fmicb.2016.01342 (PMC5014874; doi:10.3389/fmicb.2016.01342)
Supplement: Supplementary file 1 [file Data_Sheet_3.DOCX]

Supplementary Material

*Leisingera* sp. JC1, a Bacterial Isolate from Hawaiian Bobtail Squid Eggs, Produces Indigoidine and Differentially Inhibits Vibrios

Samantha M. Gromek^1,‡^, Andrea Suria^2,‡^, Matthew S. Fullmer^2^, Jillian L. Garcia^1^, Johann Peter Gogarten^2,3^, Spencer V. Nyholm^2*^, Marcy J. Balunas^1*^

^1^Division of Medicinal Chemistry, Department of Pharmaceutical Sciences, University of Connecticut, Storrs, CT, USA

^2^Department of Molecular and Cell Biology, University of Connecticut, Storrs, CT, USA

^3^Institute for Systems Genomics, University of Connecticut, Storrs, CT, USA

^‡^These authors contributed equally, listed alphabetically.

*** Correspondence:**Marcy J. Balunas, Division of Medicinal Chemistry, Department of Pharmaceutical Sciences, University of Connecticut, 69 N. Eagleville Rd. Unit 3092, Storrs, CT 06269, USA
[marcy.balunas@uconn.edu](mailto:marcy.balunas@uconn.edu)

Spencer V. Nyholm, Department of Molecular and Cell Biology, University of Connecticut, 91 N. Eagleville Rd. Unit 3125, Storrs, CT 06269, USA

[spencer.nyholm@uconn.edu](mailto:spencer.nyholm@uconn.edu)


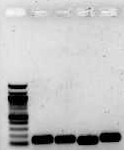


1500 bp

500 bp

300 bp

100 bp

***rpoB igiC igiD igiR***

**Supplemental Figure 1. Indigoidine biosynthesis genes *igiCDR* detected in *Leisingera* sp. JC1 DNA.** The RNA polymerase β subunit, *rpoB,* was used as a positive control for bacterial DNA. Expected product sizes: *rpoB* = 150 bp; *igiC* = 140 bp; *igiD* = 120 bp; *igiR* = 155 bp.


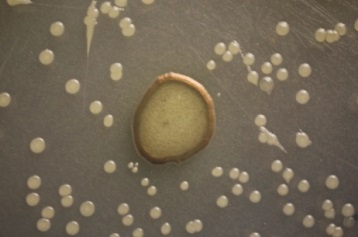

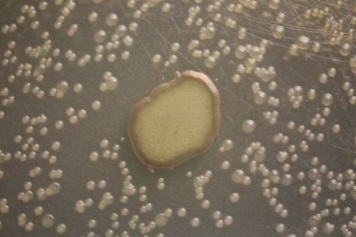

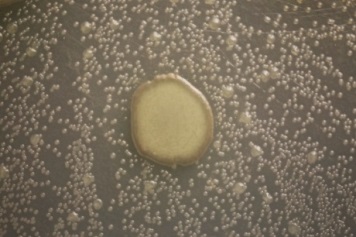

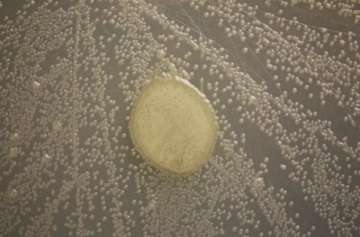

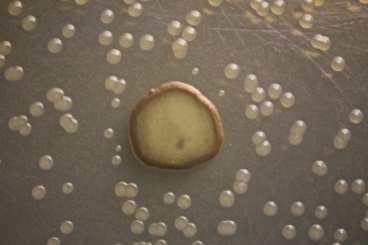

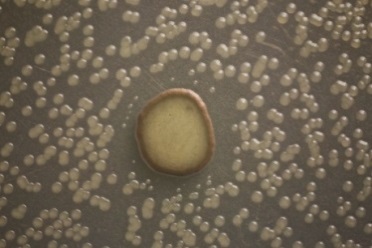

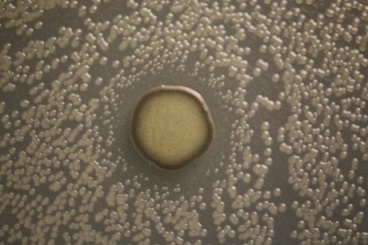

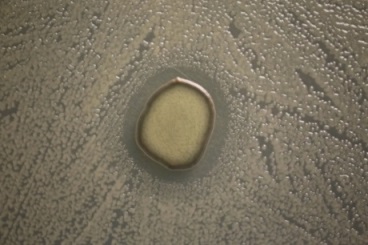

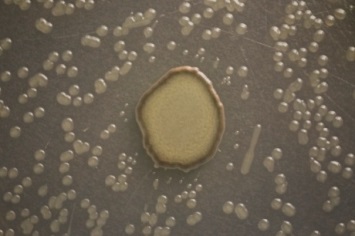

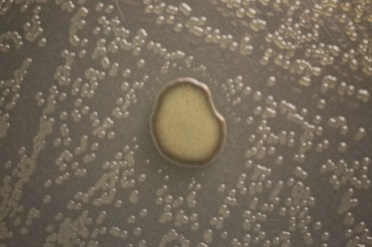

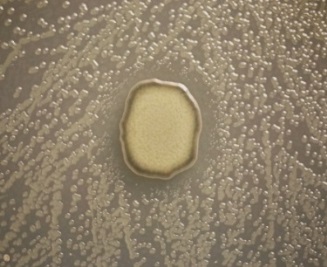

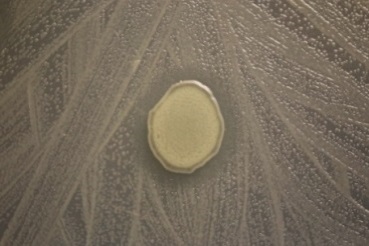

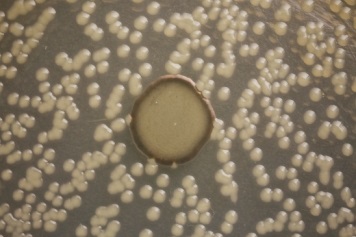

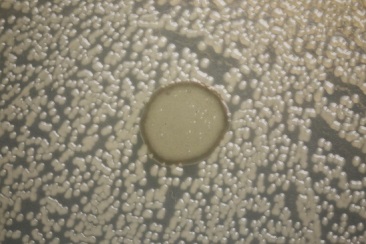

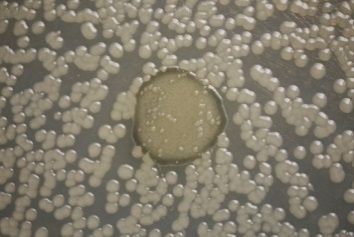

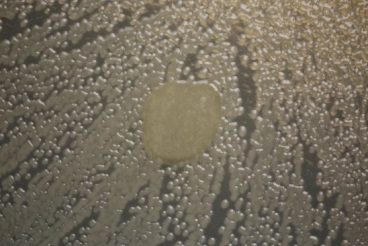


**A**

**B**

**C**

**D**

**E**

**F**

**G**

**H**

**I**

**J**

**K**

**L**

**M**

**N**

**O**

**P**

**Supplemental Figure 2. ZOI assay images for all dilutions.** *Photobacterium leiognathi* at (A) 10^4^ CFU/ml, (B) 10^5^ CFU/ml, (C) 10^6^ CFU/ml, and (D) 10^7^ CFU/ml. *Vibrio anguillarum* at (E) 10^4^ CFU/ml, (F) 10^5^ CFU/ml, (G) 10^6^ CFU/ml, and (H) 10^7^ CFU/ml. *Vibrio fischeri* at (I) 10^4^ CFU/ml, (J) 10^5^ CFU/ml, (K) 10^6^ CFU/ml, and (L) 10^7^ CFU/ml. *Vibrio harveyi* at (M) 10^5^ CFU/ml and (N) 10^6^ CFU/ml. *Vibrio parahaemolyticus* at (O) 10^5^ CFU/ml and (P) 10^6^ CFU/ml. Scale bars, 5 mm.


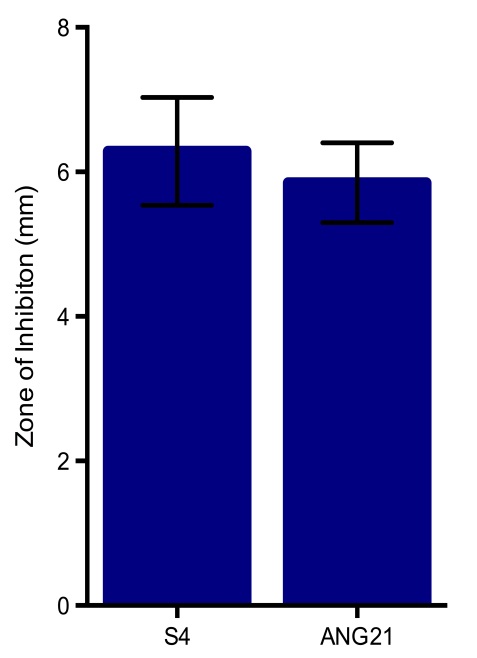

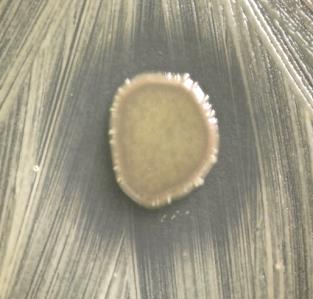

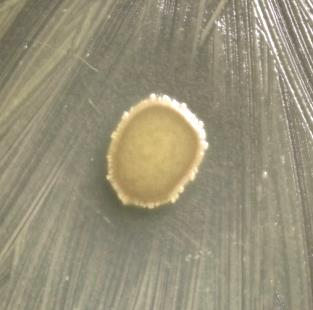


**A**

**B**

**C**

**Supplemental Figure 3. *Leisingera* sp. JC1 inhibits ANG isolates *Ruegeria* sp. ANG-S4 and *Muricauda* sp. ANG21.** (A) JC1 had an average zone of inhibition (ZOI) of 6.3 mm (±0.7) against *Ruegeria* sp. S4, and an average ZOI of 5.9 mm (±0.6) against *Muricauda* sp. ANG21. Representative images, taken 4 days after plating, of (B) *Ruegeria* sp. ANG-S4 and (C) *Muricauda* sp. ANG21. Scale bars, 5 mm.

**
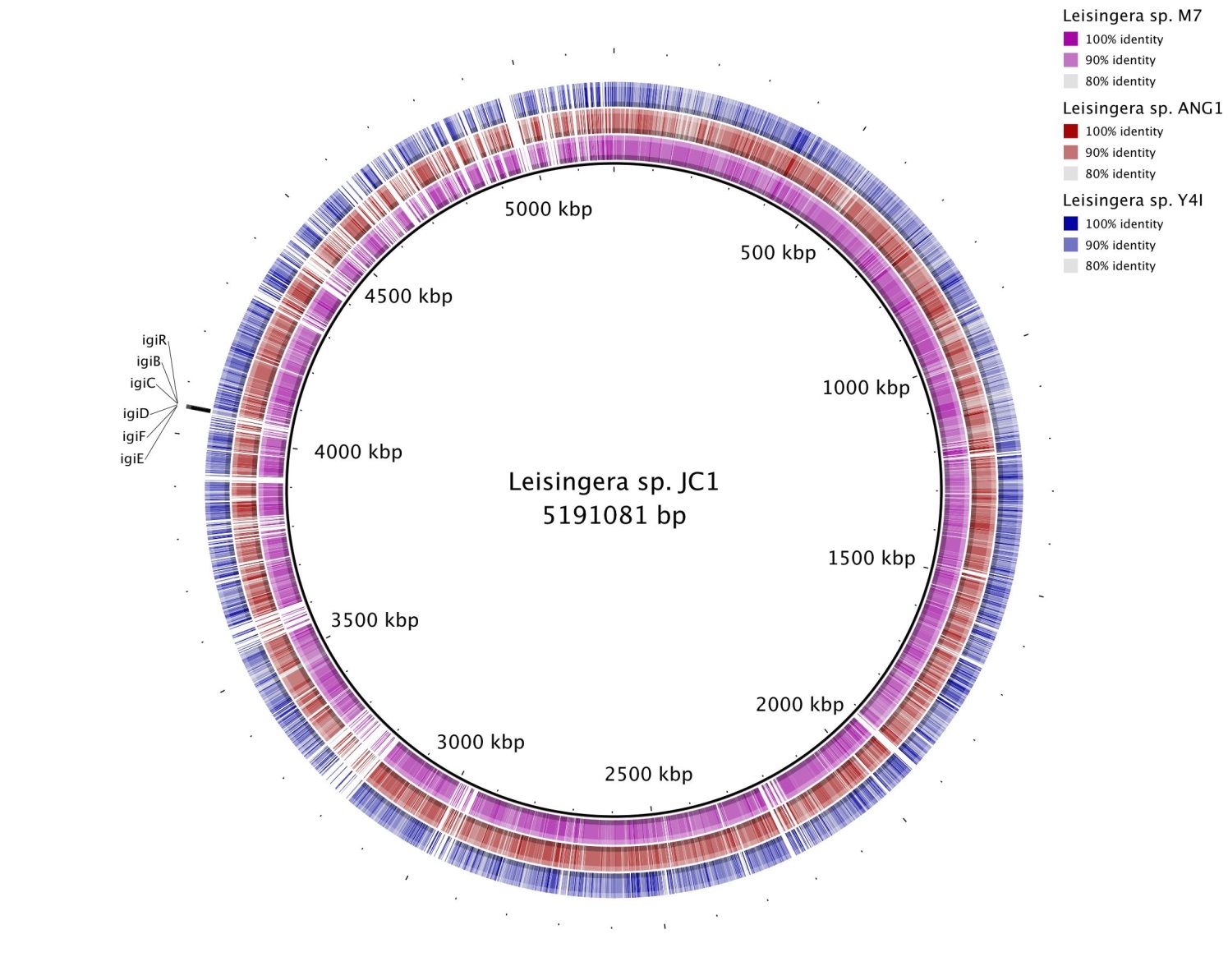
**

**Supplemental Figure 4. Genomic comparison of *Leisingera* sp. JC1 with select *Leisingera* species.** BLAST comparison of draft genomes of *Leisingera* sp. JC1 with *Leisingera* sp. ANG-M7 (purple inner ring), *Leisingera* sp. ANG1 (red middle ring), and *Leisingera* sp. Y4I (blue outer ring). Location of the indigoidine operon (*igiRBCDFE*) is called out.

**
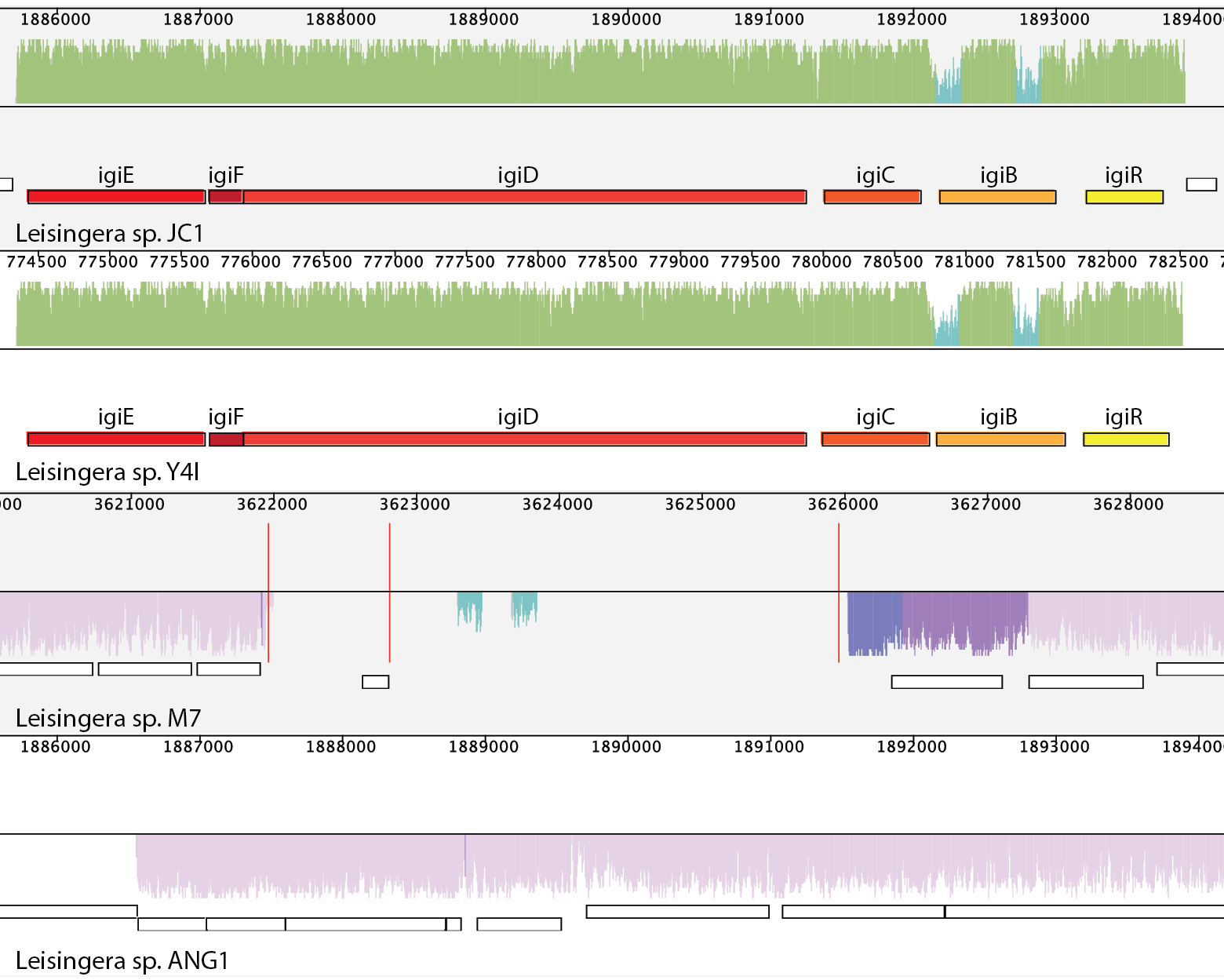
**

**Supplemental Figure 5. Genomic comparison of the *Leisingera* sp. JC1 indigoidine operon.** Comparison of JC1 indigoidine operon to the operon in *Leisingera* sp. Y4I (top), and to the genomic region in *Leisingera* sp. M7 (middle) and *Leisingera* sp. ANG1 (bottom). The JC1 operon hits to the Y4I operon (as also shown in Table 2). There are no hits when compared to M7 and ANG1, indicating the indigoidine operon is not present in these genomes.

**Supplemental Table 1. Primers designed for the detection of indigoidine biosynthesis genes in the *Leisingera* sp. JC1 genome.**

| Gene | Primer Sequence |
| --- | --- |
| *igiC* Forward | 5’ - CGGCAGCAGCGGCAAAATCC - 3’ |
| *igiC* Reverse | 5’ – CGGACGGACGCCGTCAAACA – 3’ |
| *igiD* Forward | 5’ – GGACGGGTCGCGGAATAGCG – 3’ |
| *igiD* Reverse | 5’ – CAGCTGGAGCAGGCAGGCAG – 3’ |
| *igiR* Forward | 5’ – ACCCGCTTTCCAACCGCTCG – 3’ |
| *igiR* Reverse | 5’ – CCTCCGCGCACTGTTGCTGT – 3’ |

**Supplemental Table 2. Whole genome comparisons.** Average nucleotide identities (ANI) are presented in the upper triangle and *in silico* DNA-DNA hybridization (*is*DDH) estimates are presented in the lower triangle. ANI and *is*DDH values above the usual cutoffs for species distinction are colored in maroon (ANI, 96%) and red (*is*DDH, 70%).


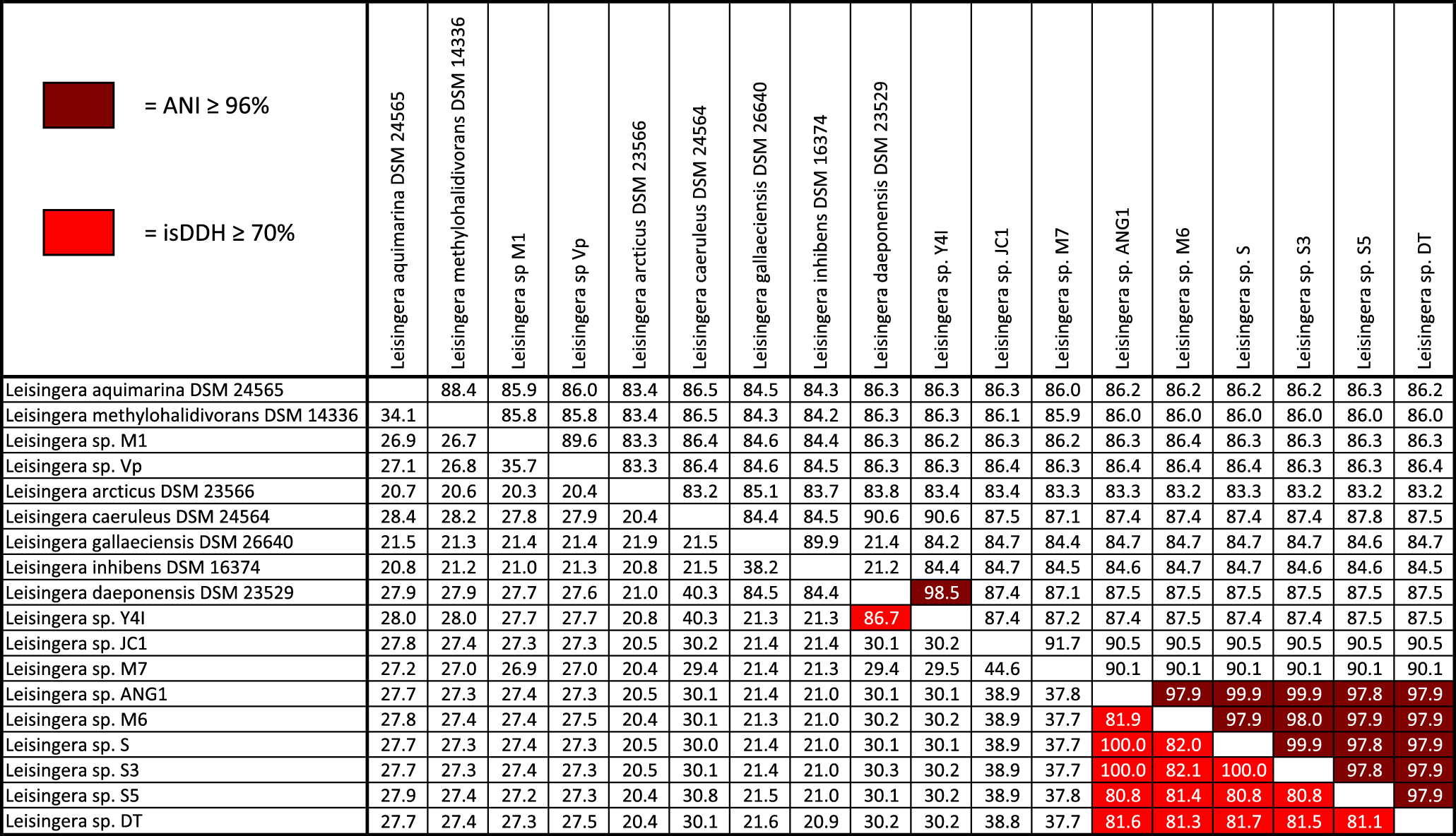


**Supplemental Table 3. Bacterial strains used in this study.**

| **Strain** | **Characteristics** | **Source** |
| --- | --- | --- |
| *Leisingera* sp. JC1 | Isolate from *E. scolopes* egg jelly coats | This study |
| *Agrobacterium tumefaciens* NTL4 pZLR4 | Reporter strain for detection of HSLs | Singh and Greenstein (2006) (Courtesy of Dr. Stephen Farrand, University of Illinois) |
| *Leisingera* sp. ANG1 | Isolate from *E. scolopes* ANG | Collins and Nyholm (2011) |
| *Ruegeria* sp. ANG-S4 | Isolate from *E. scolopes* ANG | Collins et al., (2015) |
| *Ruegeria* sp. ANG-R | Isolate from *E. scolopes* ANG | Collins et al., (2015) |
| *Leisingera* sp. ANG-Vp | Isolate from *E. scolopes* ANG | Collins et al., (2015) |
| *Leisingera* sp. ANG-M1 | Isolate from *E. scolopes* ANG | Collins et al., (2015) |
| *Leisingera* sp. ANG-DT | Isolate from *E. scolopes* ANG | Collins et al., (2015) |
| *Leisingera* sp. ANG-S | Isolate from *E. scolopes* ANG | Collins et al., (2015) |
| *Leisingera* sp. ANG-S3 | Isolate from *E. scolopes* ANG | Collins et al., (2015) |
| *Leisingera* sp. ANG-M6 | Isolate from *E. scolopes* ANG | Collins et al., (2015) |
| *Leisingera* sp. ANG-S5 | Isolate from *E. scolopes* ANG | Collins et al., (2015) |
| *Leisingera* sp. ANG-M7 | Isolate from *E. scolopes* ANG | Collins et al., (2015) |
| *Tateyamaria* sp. ANG-S1 | Isolate from *E. scolopes* ANG | Collins et al., (2015) |
| *Muricauda* sp. ANG21 | Isolate from *E. scolopes* ANG | This study |
| *Vibrio fischeri* ES114 | Isolate from *E. scolopes* light organ | Boettcher and Ruby (1990) |
| *Vibrio harveyi* B392 | Seawater isolate | Reichelt and Baumann (1973) |
| *Vibrio anguillarum* 775 | Isolate from *Oncorhynchus kisutch* | Crosa et al., (1977)  (Courtesy of Dr. Joerg Graf, University of Connecticut) |
| *Vibrio parahaemolyticus* KNH1 | Seawater isolate | Nyholm et al., (2000) |
| *Photobacterium leiognathi* KNH6 | Seawater isolate | Stabb and Ruby (2002) |

**References:**

Boettcher, K. J., and Ruby, E. G. (1990). Depressed light emission by symbiotic *Vibrio fischeri* of the sepiolid squid *Eupryma scolopes*. *J. Bacteriol.* 172, 3701–3706.

Collins, A. J., Fullmer, M. S., Gogarten, J. P., and Nyholm, S. V. (2015). Comparative genomics of *Roseobacter* clade bacteria isolated from the accessory nidamental gland of *Euprymna scolopes*. *Front. Microbiol.* 6:123. doi: 10.3389/fmicb.2015.00123

Collins, A. J., and Nyholm, S. V. (2011). Draft genome of *Phaeobacter gallaeciensis* ANG1, a dominant member of the accessory nidamental gland of *Euprymna scolopes*. *J. Bacteriol.* 193, 3397–3398. doi: 10.1128/JB.05139-11

Crosa, J. H., Schiewe, M. H., and Falkow, S. (1977). Evidence for plasmid contribution to the virulence of the fish pathogen *Vibrio anguillarum*. *Infect. Immun.* 18, 509–513.

Nyholm, S. V., Stabb, E. V., Ruby, E. G., and Mcfall-Ngai, M. J. (2000). Establishment of an animal-bacterial association: recruiting symbiotic vibrios from the environment. *Proc. Natl. Acad. Sci. U.S.A.* 97, 10231–10235. doi: 10.1073/pnas.97.18.10231

Reichelt, J. L., and Baumann, P. (1973). Taxonomy of the marine, luminous bacteria. *Archiv. Mikrobiol.* 94, 283–330. doi: 10.1007/BF00769027

Singh, M., and Greenstein, M. (2006). A simple, rapid, sensitive method detecting homoserine lactone (HSL)-related compounds in microbial extracts. *J. Microbiol. Methods* 65, 32–37. doi: 10.1016/j.mimet.2005.06.003

Stabb, E. V., and Ruby, E. G. (2002). RP4-based plasmids for conjugation between *Escherichia coli* and members of the vibrionaceae. *Methods Enzymol.* 358, 413–426. doi: 10.1016/S0076-6879(02)58106-4

**Supplemental Table 4. Detailed antiSMASH results for *Leisingera* sp. JC1.**

| Cluster | Type | From – To (bp) | Top homologous gene clusters |
| --- | --- | --- | --- |
| 1 | Homoserine lactone | (Scaffold 1.1)  332,333 – 352,974 | 43% sim to *Leisingera* sp. ANG1  39% sim to *Leisingera* sp. ANG-VP  41% sim to *Leisingera* sp. ANG-M7 |
| 2 | Bacteriocin | (Scaffold 14.1)  57,539 – 68,393 | 29% sim to *Leisingera* sp. ANG-M7  29% sim to *Leisingera* sp. ANG-DT  29% sim to *Leisingera* sp. ANG-M6 |
| 3 | Other KS | (Scaffold 17.1)  19,628 – 60,623 | 12% sim to *Citreicella* sp. SE45  12% sim to *Shinella* sp. DD12 SHLA 2c  12% sim to *Brachybacterium paraconglomeratum* |
| 4 | T1 PKS | (Scaffold 24.1)  1 - 24,550 | 45% sim to *Leisingera* sp. ANG-M7  45% sim to *Leisingera* sp. ANG-S3  45% sim to *Leisingera* sp. ANG1 |
| 5 | Other | (Scaffold 34.1)  13,994 – 41,877 | 17% sim to *Leisingera* sp. Y4I  14% sim to *Vogesella indigofera*  11% sim to *Gordonia soli* NBRC 108243 |
| 6 | Siderophore | (Scaffold 63.1)  2,810 – 14,229 | 18% sim to *Mesorhizobium* sp. L48C026A00  18% sim to *Rhizobium trpici* CIAT 899 plasmid pRtrCIAT899c  16% sim to *Acidovorax radices* N35 |
| 7 | Other | (Scaffold 79.1)  1 – 9,934 | 16% sim to *Leisingera* sp. ANG-M7  16% sim to *Leisingera* sp. ANG-M6  16% sim to *Leisingera* sp. ANG1 |
| 8 | Siderophore | (Scaffold 99.1)  1 – 3,224 | 7% sim to *Leisingera* sp. ANG1  7% sim to *Leisingera* sp. ANG-S  7% sim to *Leisingera* sp. ANG-S3 |
| 9 | Siderophore | (Scaffold 114.1)  1 – 1,268 | None found |
